# ﻿First record of the genus *Aspilota* Foerster, 1863 in Argentina (Hymenoptera, Braconidae, Alysiinae), with the description of the new species *Aspilotamurieli* sp. nov. and a key to the Neotropical taxa

**DOI:** 10.3897/zookeys.1236.150605

**Published:** 2025-04-24

**Authors:** Francisco Javier Peris-Felipo, Camila Noemí Villar, Sofía Belén Forte, Joel Nazareno Lentini, María del Pilar Medialdea, Analí Bustos, Federico Pandol-Avalos, Ana Lia Gayan-Quijano, Santiago L. Poggio, Sergey Belokobylskij, Mariano Devoto

**Affiliations:** 1 Bleichestrasse 15, CH-4058 Basel, Switzerland Syngenta Crop Protection AG Basel Switzerland; 2 Syngenta Crop Protection AG, Rosentalstrasse 67, CH-4058 Basel, Switzerland Unaffiliated Basel Switzerland; 3 Universidad de Buenos Aires, Facultad de Agronomía, Cátedra de Botánica General, C. A. de Buenos Aires, Argentina Universidad de Buenos Aires Buenos Aires Argentina; 4 Syngenta Agro S.A., Av. del Libertador 1638, B1638 BGQ, Vicente Lopez, Buenos Aires, Argentina Syngenta Agro S.A. Buenos Aires Argentina; 5 Instituto de Investigaciones Fisiológicas y Ecológicas Vinculadas a la Agricultura (IFEVA), Facultad de Agronomía, C. A. de Buenos Aires, Argentina Instituto de Investigaciones Fisiológicas y Ecológicas Vinculadas a la Agricultura (IFEVA), Facultad de Agronomía C. A. de Buenos Aires Argentina; 6 Universidad de Buenos Aires, Facultad de Agronomía, Cátedra de Producción Vegetal, C. A. de Buenos Aires, Argentina Universidad de Buenos Aires C. A. de Buenos Aires Argentina; 7 Zoological Institute of the Russian Academy of Sciences, St. Petersburg, 199034, Russia Zoological Institute of the Russian Academy of Sciences St. Petersburg Russia; 8 Consejo Nacional de Investigaciones Científicas y Técnicas (CONICET), Buenos Aires, Argentina Consejo Nacional de Investigaciones Científicas y Técnicas (CONICET) Buenos Aires Argentina

**Keywords:** Alysiinae, *Aspilota*-group, diagnosis, identification key, parasitoid, South America

## Abstract

A new species of *Aspilota* without a mesoscutal pit, *A.murieli* Peris-Felipo, **sp. nov.**, is described and illustrated from Argentina. The genus *Aspilota* Foerster, 1863 is recorded from Argentina for the first time. A key to the Neotropical species of *Aspilota* is provided.

## ﻿Introduction

The genus *Aspilota* Foerster, 1863 is distinguished from other members of the subtribe Aspilotina by three key features: paraclypeal fovea extended to the inner eye margin, a closed distal brachial (first subdiscal) cell, and the presence of the vein cuqu1 (2-SR) in the fore wing (van Achterberg 1988; Peris-Felipo and Belokobylskij 2016; Peris-Felipo et al. 2025).

*Aspilota* species are primarily endoparasitoids of Diptera, Cyclorrhapha, with a focus on the family Phoridae. Records of hosts from other families such as Tephritidae, Anthomyiidae, or Sarcophagidae (Yu et al. 2016) are considered questionable and require further investigation (van Achterberg 1988). The genus comprises approximately 250 species described from nearly all zoogeographic regions.

Current knowledge of *Aspilota* in the Neotropical region is very limited. Prior to this study, only three species had been documented in this realm: *Aspilotastigmalis* Papp, 2012 from Colombia, and *A.nemostigma* Spinola, 1851 and *A.pulchella* Spinola, 1851 from Chile. Unfortunately, our study excludes the Chilean species due to two significant challenges. Firstly, we were unable to find the place of preservation of the type specimens for their study and revision. Secondly, the original descriptions of these species are extremely vague. These factors combined made it difficult to identify and select the necessary diagnostic characteristics needed for accurate identification in our research.

In this paper, the genus *Aspilota* is recorded for the first time from Argentina. *Aspilotamurieli* sp. nov., characterized by the small size of the upper tooth of the mandible, is described and illustrated. Moreover, an identification key of the available Neotropical species is provided.

## ﻿Materials and methods

**Figure 1.**
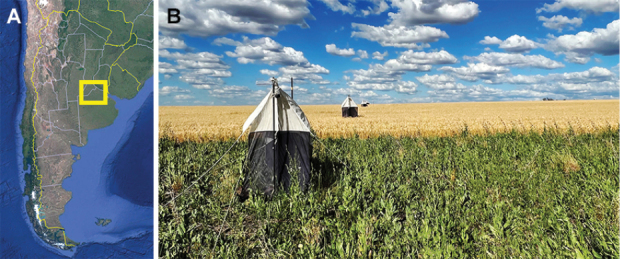
**A** Farms location in Argentina **B** View of a wheatfield with Malaise traps installed.

This study was conducted in the Rolling Pampas of Argentina (Fig. 1A), a region characterized by a temperate climate (Cfa/Cfb in the Köppen climate classification) (Soriano et al. 1991; Bianchi and Cravero 2010). The area experiences distinct seasonal variations, with warm to hot summers and cold winters. Average annual temperatures range from 13 °C to 17 °C, with summer highs exceeding 30 °C and winter lows occasionally dropping below freezing. The region is notable for its significant diurnal temperature fluctuations, particularly during summer. Annual precipitation in the Rolling Pampas varies between 800 and 1,200 mm, with the majority occurring in summer (Soriano et al. 1991; Bianchi and Cravero 2010).

The research encompassed 12 fields distributed in the Baradero, Solis, Teodelina and Vedia Districts from Argentina (Fig. 1A). The selected fields, with an average size of 77.25 ha (ranging from 40 to 213 ha), were managed under a rotational cropping system that alternated between corn, soybean, and wheat. Each field was equipped with 10 Malaise traps strategically placed at 10, 50, 100, and 150 m from the field edge (Fig. 1B). Two additional traps were set within 10 × 100 m strips of seminatural vegetation at the field margins. One of these strips was experimentally enhanced with a multifunctional floral margin to promote biodiversity, while the other served as an untreated control.

Trapping was conducted only when crops were present, with traps removed before harvest and reinstalled shortly after sowing. No data was collected during fallow periods. The study spanned two years (July 2022 to April 2024). In the first year (July 2022 to May 2023), traps operated continuously, with bi-monthly collections. The second year (August 2023 to April 2024) involved 15-day trapping periods each month.

Collected specimens were preserved in 70% alcohol and later identified to species. External morphology was examined using a ZEISS Discovery V8 stereomicroscope, with several specimens dissected and slide-mounted in Berlese medium for detailed analysis.

For the terminology of the morphological features, sculpture, and measurements, see Peris-Felipo et al. (2014); for wing venation nomenclature, see Peris-Felipo et al. (2014) and van Achterberg (1993). Following abbreviations have been used: **POL** (post-ocellar line, shortest distance between inner margins of lateral ocelli), **OOL** (ocular-ocellar line, shortest distance between outer margin of lateral ocellus and inner margin of eye), and **OD** (maximum diameter of ocellus). The species was identified by reviewing the description of the Neotropical *Aspilota* species (Spinola 1851; Fischer 1971; Papp 2012) due to the absence of a key to New World species.

For the molecular methods, the DNA from each sample was isolated using the Quick-DNA Microprep Plus kit (Zymo Research), specifically optimized for small tissue samples, strictly following the manufacturer’s instructions. The DNA was eluted in a final volume of 12 µL. DNA concentration was quantified using the Qubit High Sensitivity dsDNA Assay (Thermo Fisher Scientific). For PCR amplification, a 650-bp fragment from the 5′ region of CO1 was amplified using the LepF1 and LepR1 primers (Hebert et al. 2003, 2004; Park et al. 2010). Moreover, a fragment of around 666 bp of the 28S rRNA gene was amplified using D2-3665F and D3-4283R primers (Mardulyn and Whitfield 1999). PCRs were carried out in a final volume of 10 μL, containing 2.9 μL of template DNA, 0.5 μM of the primers, 5 μL of Supreme NZYTaq 2x Green Master Mix (NZYTech), CES 1X (Ralser et al. 2006), and ultrapure water up to 10 μL. The reaction mixture was incubated as follows: an initial denaturation step at 95 °C for 5 min, followed by 35 cycles of 95 °C for 30 s, 48.5 °C (LepF1 and LepR1) or 52.7 °C (D2-3665F and D3-4283R) for 60 s, 72 °C for 45 s, and a final extension step at 72 °C for 7 min. The PCR products were bi-directionally sequenced on an ABI 3730xl DNA Analyzer (Applied Biosystems, USA), with the same primers as those used in the PCR amplification. The amplification products were purified using magnetic beads (MagBind, Omega-Bio-tek) prior to sequencing.

The material was imaged using Keyence^®^ VHX-2000 Digital Microscope and post processed in Adobe Photoshop^®^. The specimens are deposited in the Entomological collection of the Bernardino Rivadavia Natural Sciences Argentine Museum (Buenos Aires, Argentina; **MACN-En**), the Naturhistorisches Museum Basel (Basel, Switzerland; **NMB**), the Zoological Institute RAS (St Petersburg, Russia; **ZISP**), and the F.J. Peris-Felipo Private Entomological Collection (Basel, Switzerland; **PFEC**).

## ﻿Taxonomic part

### ﻿Order Hymenoptera Linnaeus, 1758

**Family Braconidae Nees, 1811**

**Subfamily Alysiinae Leach, 1815**

**Genus *Aspilota* Foerster, 1863**

#### ﻿ Aspilota murieli

Taxon classificationAnimaliaHymenopteraBraconidae

Peris-Felipo sp. nov.

06E7BF65-7BBB-5A00-A83F-17FFC7C8244B

https://zoobank.org/3D60F943-4189-4308-AE38-D9F87E897DB5

Figs 2 , 3

##### Type material.

***Holotype***: Argentina • ♀; Buenos Aires Province, Partido de Baradero; 33°55'13"S, 59°37'26"W; 24 m; 10.xi.2022; Malaise trap (Peris-Felipo leg.) (MACN-En).

***Paratypes***: Argentina • 7 ♀♀; same location than holotype but: 2♀♀; 8.ix.2022; 33°56'16"S, 59°37'4"W; 16 m (MACN-En; ZISP); 1 ♀; 5.x.2022; 33°55'16"S 59°37'42"W; 16 m (MACN-En) • 1 ♀; 24.x.2022; 33°55'13"S, 59°37'26"W; 99 m (NMB) • 1 ♀; 23.xi.2022; 33°56'15"S, 59°36'40"W; 19 m (NMB) • 2 ♀♀; 3.x.2023; 33°55'16"S, 59°37'42"W; 18 m (PFEC) • 1 ♀; Buenos Aires Province, Partido de Solis; 5.x.2022; 34°12'10"S, 59°13'22"W; 31 m; Malaise trap (Peris-Felipo leg.) (MACN-En) • 1 ♀; Buenos Aires Province, Partido de Solis; 7.ii.2024; 34°11'29"S, 59°13'44"W; 29 m; Malaise trap (Peris-Felipo leg.) (PFEC) • 2 ♀♀; Santa Fé Province, Partido de Teodelina; 13.x.2022; 34°06'18"S, 61°27'25"W; 97 m; Malaise trap (Peris-Felipo leg.) (PFEC; ZISP) • 1 ♀; Buenos Aires Province, Partido de Vedia; 12.x.2022; 34°27'35"S, 61°48'46"W; 99 m; Malaise trap (Peris-Felipo leg.) (MACN-En) • 1 ♀; Buenos Aires Province, Partido de Vedia; 31.x.2022; 34°29'10"S, 61°47'12"W; 99 m; Malaise trap (Peris-Felipo leg.) (MACN-En) • 1 ♀, 1 ♂, Buenos Aires Province, Partido de Vedia; 10.i.2024; 34°29'10"S, 61°47'12"W; 99 m; Malaise trap (Peris-Felipo leg.) (PFEC) • 1 ♀, Buenos Aires Province, Partido de Vedia, 18.vii.2024; 34°29'25"S, 61°47'45"W; 99 m; Malaise trap (Peris-Felipo leg.) (NMB).

##### Description.

**Female** (holotype). ***Length*.** Body 1.4 mm; fore wing 1.6 mm; hind wing 1.2 mm.

**Figure 2.**
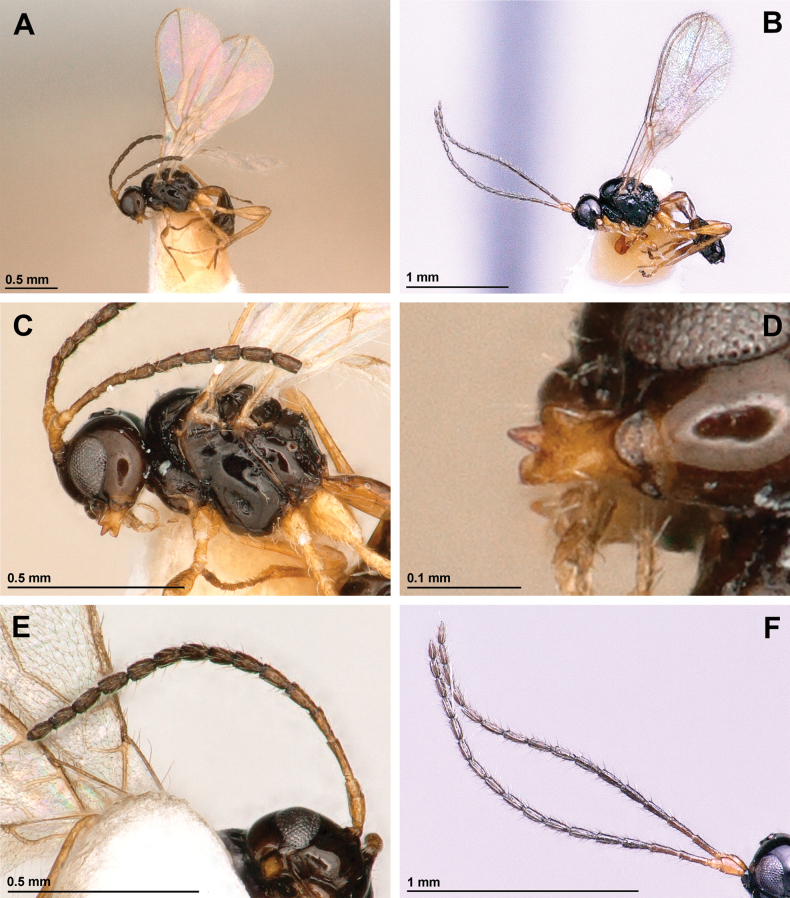
*Aspilotamurieli* sp. nov. **A, C–E** female, holotype **B, F** male, paratype **A, B** habitus, lateral view **C** head and mesosoma, lateral view **D** mandible **D, E** antenna.

**Figure 3.**
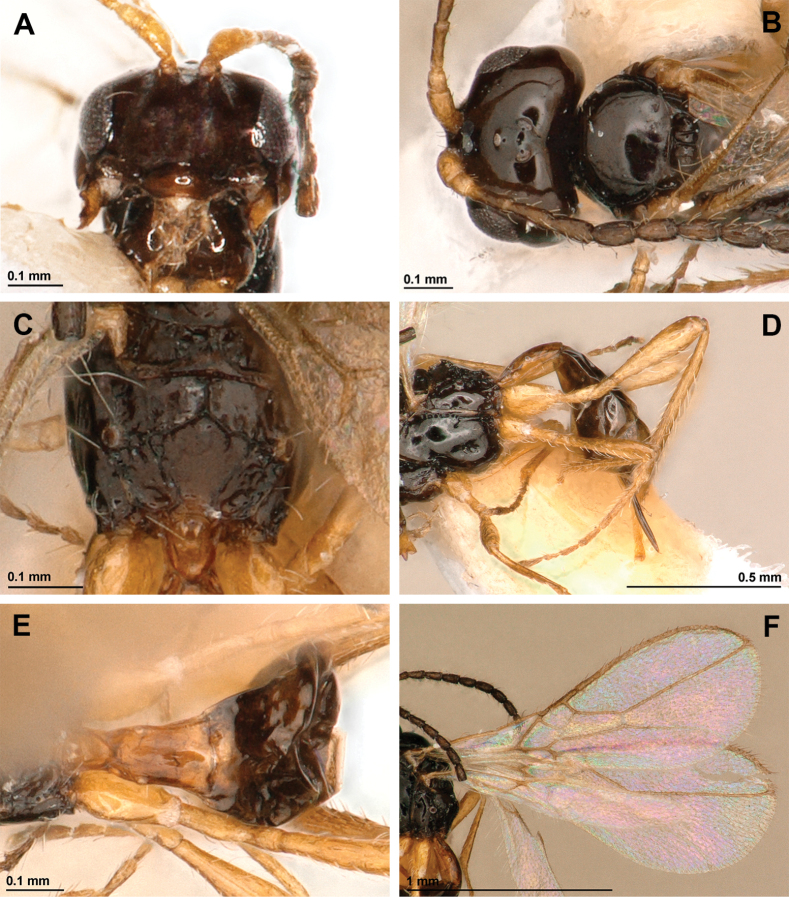
*Aspilotamurieli* sp. nov. **A–F** female, holotype **A** head, front view **B** head, dorsal view **C** propodeum, dorsal view **D** hind leg, metasoma and ovipositor, lateral view **E** first metasomal tergite **F** wings.

***Head*.** In dorsal view, 1.85 × as wide as its median long, 1.5 × as wide as mesoscutum, smooth, with temple rounded behind eyes (Fig. 3B). Eye in lateral view 1.6 × as high as wide and 0.9 × as wide as temple medially (Fig. 2C). POL 1.6 × OD; OOL ~3.0 × OD (Fig. 3B). Face 1.7 × as wide as high; inner margins of eyes subparallel (Fig. 3A). Clypeus 2.3 × as wide as high, slightly curved ventrally (Fig. 3A). Paraclypeal fovea reaching inner margin of eye (Fig. 3A). Mandible tridentate, weakly widened towards apex, 1.3 × as long as its maximum width (Fig. 2D). Upper tooth distinctly shorter than lower tooth, small and rounded; middle tooth rather long and narrow, longer than lower tooth, acuminate apically; lower tooth widest, obtuse, subrounded distally, weakly curved downwards (Fig. 2D). Antenna (Fig. 2E) 15-segmented, 0.8 × as long as body. Scape 2.0 × longer than pedicel. First flagellar segment 3.7 × as long as its maximum width, 1.2 × as long as second segment. Second flagellar segment 2.6 × as long as its maximum width; third to eleventh segments 1.7–1.8 × as long as their maximum width, 12^th^ segment 1.45×, and 15^th^ (apical) segment 2.0 × as long as their wide accordingly (Fig. 2E).

***Mesosoma*.** In lateral view, 1.2 × as long as high (Fig. 2C). Mesoscutum (dorsal view) 0.75 × as long as its maximum width, smooth, without setae along tracks of notauli (Fig. 3B). Notauli mainly absent on horizontal surface of mesoscutum (Fig. 3B). Mesoscutal pit absent (Fig. 3B). Prescutellar depression smooth, with three carinae (Fig. 3B). Precoxal sulcus present, crenulate, short, not reaching anterior and posterior margins of mesopleuron (Fig. 2C). Posterior mesopleural furrow crenulate in upper half, smooth in lower half (Fig. 2C). Propodeum largely smooth, with pentagonal areola delineated by distinct carinae (Fig. 3C). Propodeal spiracles weakly enlarged, its diameter 0.5 × distance from spiracle to anterior margin of propodeum. (Fig. 3C).

***Wings*** (Fig. 3F). Length of fore wing 2.5 × as long as its maximum width. Radial (marginal) cell ending at apex of wing, 3.7 × as long as its maximum width. Vein r2 (3-SR) 2.3 × as long as vein cuqu1 (2-SR); vein r3 (SR1) 3.1 × as long as vein r2 (3-SR). Nervulus (cu-a) distinctly postfurcal. Brachial (first subdiscal) cell closed distally, 2.6 × as long as its maximum width. Hind wing 6.7 × as long as its maximum width.

***Legs*** (Fig. 3D). Hind femur subclaviform, 3.8 × as long as its maximum width. Hind tibia weakly widened towards apex, 9.2 × as long as its maximum subapical width, 1.05 × as long as its hind tarsus. First segment of hind tarsus 2.4 × as long as second segment.

***Metasoma*.** First tergite long, slightly widened towards apex, 2.0 × as long as its apical width, completely smooth (Fig. 3E). Ovipositor sheath (Fig. 3D) 1.3 × as long as first tergite, 0.4 × as long as metasoma, 0.5 × as long as hind femur, 0.3 × as long as fore wing.

***Colour*.** Body and antenna dark brown. Mandibles, palpi, pterostigma and legs yellowish brown. First metasomal tergite paler than second and third tergites. Wings hyaline.

***Variation*.** Body length 1.3–1.7 mm; fore wing length 1.5–1.9 mm; hind wing length 1.1–1.5 mm. Face 1.60–1.75 × as wide as high. Mandible 1.3–1.4 × as long as its maximum width. Antenna 14–16-segmented. First flagellar segment 3.6–3.7 × as long as its maximum width. Sixth flagellar segment 1.6–1.8 × as long as its maximum width. Vein r3 (SR1) 2.9–3.2 × as long as vein r2 (3-SR). Hind femur 3.65–3.80 × as long as its maximum width.

**Male** (Fig. 2B, F). Body length 1.2 mm; fore wing length 1.9 mm; hind wing length 1.2 mm. Antenna slender, 18-segmented, about as long as body. First flagellar segment 3.8 × as long as its maximum width. Second flagellar segment 2.6 × as long as its maximum width. Third and fourth segments 3.15×, 5^th^–11^th^ segments 2.8×, 12^th^–14^th^ segments 2.5×, and 15^th^ segment 3.0 × as long as its maximum width. Vein r3 (SR1) 3.0 × as long as vein r2 (3-SR). Otherwise similar to female.

##### DNA sequence data.

Sequences obtained as part of this study are deposited in GenBank, accession numbers PV097239 and PV097240.

##### Etymology.

This species is named in honour of Julio Muriel, for his motivational influence, visionary inspiration and his key role as the driving force behind the success of this project.

##### Comparative diagnosis.

This new species is similar to *A.stigmalis* from Colombia (Papp 2012) (Neotropical) and *A.spiracularis* from Mexico (Nearctic) (Fischer 1970). *Aspilotamurieli* sp. nov. differs from *A.stigmalis* on having the eye in dorsal view as wide as temple medially (0.8 × as wide as in *A.stigmalis*) and in lateral view 0.9 × as wide as temple medially (1.1 × in *A.stigmalis*), mandible 1.3–1.4 × as long as its maximum width (1.6 × in *A.stigmalis*), scape 2.0 × longer than pedicel (2.5 × in *A.stigmalis*), and vein r3 (SR1) 2.9–3.2 × as long as vein r2 (3-SR) (2.3 × in *A.stigmalis*). On the other hand, this new species differs from *A.spiracularis* on having the head in dorsal view 1.85 × as wide as its median long (1.33 × in *A.spiracularis*), first flagellar segment 3.6–3.7 × as long as its maximum width (5.0 × in *A.spiracularis*), vein r2 (3-SR) 2.3 × as long as vein cuqu1 (2-SR) (1.4 × in *A.spiracularis*), hind femur 3.65–3.80 × as long as its maximum width (4.0 × in *A.spiracularis*), and first metasomal tergite 2.0 × as long as its apical width (1.7 × in *A.spiracularis*).

### ﻿Key to the Neotropical species of *Aspilota*

Both Spinola’s Chilean species, *A.nemostigma* (Spinola, 1851) and *A.pulchella* (Spinola, 1851), have been excluded from the key due to the unavailablility of specimens and ambiguous original descriptions, preventing accurate identification for our study.

**Table:** 

1	Vein r2 (3-SR) 1.4 × as long as vein cuqu1 (2-SR). First flagellar segment 5.0 × as long as its maximum width. First metasomal tergite about 1.7 × as long as its apical width. [Body length 2.4 mm. Mexico]	***A.spiraculis* Fischer** ♀
–	Vein r2 (3-SR) 2.3–2.5 × as long as vein cuqu1 (2-SR). First flagellar segment 3.6–3.8 × as long as its maximum width. First metasomal tergite about 2.0 × as long as its apical width	**2**
2	Eye in dorsal view 0.8 × as wide as temple, and in lateral view 1.1 × as wide as temple medially. Mandible 1.6 × as long as its maximum width. Scape 2.5 × longer than pedicel. Vein r3 (SR1) 2.3 × as long as vein r2 (3-SR). [Body length 3.0 mm. Colombia]	***A.stigmalis* Papp** ♀
–	Eye in dorsal view as wide as temple, and in lateral view 0.9 × as wide as temple medially. Mandible 1.3–1.4 × as long as its maximum width. Scape 2.0 × longer than pedicel. Vein r3 (SR1) 2.9–3.2 × as long as vein r2 (3-SR). [Body length 1.2–1.7 mm. Argentina]	***A.murieli* Peris-Felipo, sp. nov.** ♀♂

## ﻿Discussion

Species of the genus *Aspilota* are endoparasitoids of Diptera, laying their eggs in larvae and emerging from the host. They have been recorded across multiple zoogeographical regions worldwide. However, until now, knowledge of this group in the Neotropical region was restricted to Chile and Colombia (Yu et al. 2016). This study provides the first well-documented record of the genus *Aspilota* in Argentina and includes the description of *Aspilotamurieli* sp. nov. The genetic data analysis confirms that this species is new to science, with its closest known relative being *A.angusta* Berry, 2007, previously recorded in Australia, Canada, and New Zealand. However, with a genetic similarity of only 94.92%, it is evident that *A.murieli* represents a newly identified species.

The diagnostic morphological traits of the *Aspilota* group align closely with the primary, well-established characteristics of the genus. These include the paraclypeal fovea extending to the inner eye margin, a closed distal brachial (first subdiscal) cell, and the presence of the vein cuqu1 (2-SR) in the forewing. These shared features facilitate genus identification.

The newly developed key for identifying the Neotropical *Aspilota* species, presented in this study, marks an important step toward advancing research on the biodiversity of this genus in the region.

The scarcity of data on this genus in the Neotropics may be attributed to the limited number of specialists focusing on parasitoid wasps and the general lack of entomological studies in the region. Future research on Argentine parasitoid wasps is strongly encouraged to gain a better understanding of their distribution and biology, as many of these species play a vital role in the biological control of fly pest populations.

## Supplementary Material

XML Treatment for Aspilota murieli

## ﻿Additional information

### Author ORCIDs

Francisco Javier Peris-Felipo https://orcid.org/0000-0001-9929-3277

Camila Noemí Villar https://orcid.org/0009-0008-2756-4670

Sofía Belén Forte https://orcid.org/0009-0004-7551-2120

Joel Nazareno Lentini https://orcid.org/0009-0002-7901-0633

María del Pilar Medialdea https://orcid.org/0009-0008-6214-9472

Federico Pandol-Avalos https://orcid.org/0009-0005-0458-4837

Ana Lia Gayan-Quijano https://orcid.org/0009-0001-1457-3788

Santiago L. Poggio https://orcid.org/0000-0001-9949-9240

Sergey Belokobylskij https://orcid.org/0000-0002-3646-3459

Mariano Devoto https://orcid.org/0000-0003-3098-236X

### Data availability

All of the data that support the findings of this study are available in the main text.

## References

[B1] Bianchi AR Cravero SAC (2010) Atlas Climático Digital de la República Argentina. Ediciones Instituto Nacional de Tecnología Agropecuaria, Buenos Aires, Argentina, 57 pp.

[B2] Fischer M (1971) Revision der nearktischen *Aspilota*-Arten der Sektion D. und Erganzungen zu anderen Arten-gruppen. Annalen des Naturhistorisches Museum in Wien 74: 91–127.

[B3] Hebert PD Penton EH Burns JM Janzen DH Hallwachs W (2004) Ten species in one: DNA barcoding reveals cryptic species in the neotropical skipper butterfly *Astraptesfulgerator*. Proceedings of the National Academy of Sciences of the United States of America 101: 14812–14817. 10.1073/pnas.0406166101 15465915 PMC522015

[B4] Herbert PD Cywinska A Ball SL de Waard JR (2003) Biological identifications through DNA barcodes. Proceedings of Biological Science 270(1512): 313–321. 10.1098/rspb.2002.2218 PMC1691236 12614582

[B5] Mardulyn P Whitfield JB (1999) Phylogenetic signal in the COI, 16S, and 28S genes for inferring relationships among genera of Microgastrinae (Hymenoptera; Braconidae): evidence of a high diversification rate in this group of parasitoids. Molecular Phylogenetics and Evolution 12: 282–294. 10.1006/mpev.1999.0618 10413623

[B6] Papp J (2012) Five new braconid species from Colombia (Hymenoptera, Braconidae). Journal of Hymenoptera Research 28: 67–84. 10.3897/jhr.28.2023

[B7] Park D-S Suh S-J Oh H-W Herbert PD (2010) Recovery of the mitochondrial COI barcode region in diverse Hexapoda through tRNA-based primers. BMC Genomics 11: 423. 10.1186/1471-2164-11-423 PMC2996951 20615258

[B8] Peris-Felipo FJ Belokobylskij SA (2016) First record of the genus *Dinotrema* Foerster, 1863 (Hymenoptera, Braconidae, Alysiinae) from the Neotropical region with description of four new species and a key to the New World taxa. European Journal of Taxonomy 179: 1–23. 10.5852/ejt.2016.179

[B9] Peris-Felipo FJ Belokobylskij SA Jiménez-Peydró R (2014) Revision of the Western Palaearctic species of the genus *Dinotrema* Foerster, 1862 (Hymenoptera, Braconidae, Alysiinae). Zootaxa 3885(1): 1–483. 10.11646/zootaxa.3885.1.1 25543842

[B10] Peris-Felipo FJ Santa F van Achterberg C Belokobylskij SA (2025) Review of the genera and subgenera of the subtribe Aspilotina (Hymenoptera, Braconidae, Alysiinae), with a new illustrated key. ZooKeys 1229: 133–200. 10.3897/zookeys.1229.142489 40051644 PMC11883488

[B11] Ralser M Querfurth R Warnatz H Lehrach H Yaspo M Krobitsch S (2006) An efficient and economic enhancer mix for PCR. Biochemical and Biophysical Research Communications 347: 747–751. 10.1016/j.bbrc.2006.06.151 16842759

[B12] Soriano A León RJC Sala OE Lavado R Deregibus VA Cauhepé MA Scaglia OA Velázquez CA Lemcoff JH (1991) Río de la Plata Grasslands. In: Coupland RT (Ed.) Temperate Subhumid Grasslands.Ecosystems of the World. 8, Natural Grasslands. Elsevier, Amsterdam, 367–407.

[B13] Spinola M (1851) Icneumonitos. In: Gay C (Ed.) Historia física y politica de Chile, Paris, 471–550.

[B14] van Achterberg C (1988) The genera of the *Aspilota*-group and some descriptions of fungicolous Alysiini from Netherlands (Hymenoptera: Braconidae: Alysiinae). Zoologische Verhandelingen Leiden 247: 1–88.

[B15] van Achterberg C (1993) Illustrated key to the subfamilies of the Braconidae (Hymenoptera: Ichneumonoidea). Zoologische Verhandelingen Leiden 283: 1–189.

[B16] Yu DS van Achterberg C Horstmann K (2016) . Taxapad 2016, Ichneumonoidea 2015. Taxapad, Ottawa, Ontario. [Database on flash-drive]

